# A biomimetic enzyme-linked immunosorbent assay (BELISA) for the analysis of gonadorelin by using molecularly imprinted polymer-coated microplates

**DOI:** 10.1007/s00216-021-03867-7

**Published:** 2022-01-13

**Authors:** Francesca Torrini, Laura Caponi, Andrea Bertolini, Pasquale Palladino, Francesca Cipolli, Alessandro Saba, Aldo Paolicchi, Simona Scarano, Maria Minunni

**Affiliations:** 1 Department of Chemistry ‘Ugo Schiff’, University of Florence, Sesto Fiorentino (FI), Italy; 2 Laboratory of Clinical Pathology, University Hospital of Pisa, Pisa, Italy; 3 Department of Surgical, Medical and Molecular Pathology and Critical Care Medicine, University of Pisa, Pisa, Italy

**Keywords:** Molecularly imprinted polymers, Antibody mimetics, Polydopamine, Polynorepinephrine, Enzyme-linked immunosorbent assay, Gonadotropin-releasing hormone

## Abstract

An original biomimetic enzyme-linked immunoassay (BELISA) to target the small peptide hormone gonadorelin is presented. This peptide has been recently listed among the substances banned in sports by the World Antidoping Agency (WADA) since its misuse by male athletes triggers testosterone increase. Hence, in response to this emerging issue in anti-doping controls, we proposed BELISA which involves the growth of a polynorepinephrine (PNE)–based molecularly imprinted polymer (MIP) directly on microwells. PNE, a polydopamine (PDA) analog, has recently displayed impressive performances when it was exploited for MIP preparation, giving even better results than PDA. Gonadorelin quantification was accomplished via a colorimetric indirect competitive bioassay involving the competition between biotinylated gonadorelin linked to the signal reporter and the unlabeled analyte. These compete for the same MIP binding sites resulting in an inverse correlation between gonadorelin concentration and the output color signal (*λ* = 450 nm). A detection limit of 277 pmol L^−1^ was achieved with very good reproducibility in standard solutions (_av_CV% = 4.07%) and in urine samples (_av_CV% = 5.24%). The selectivity of the assay resulted adequate for biological specimens and non-specific control peptides. In addition, the analytical figures of merit were successfully validated by mass spectrometry, the reference anti-doping benchtop platform for the analyte. BELISA was aimed to open real perspectives for PNE-based MIPs as alternatives to antibodies, especially when the target analyte is a poorly or non-immunogenic small molecule, such as gonadorelin.

**Graphical abstract:**

**Figure:**
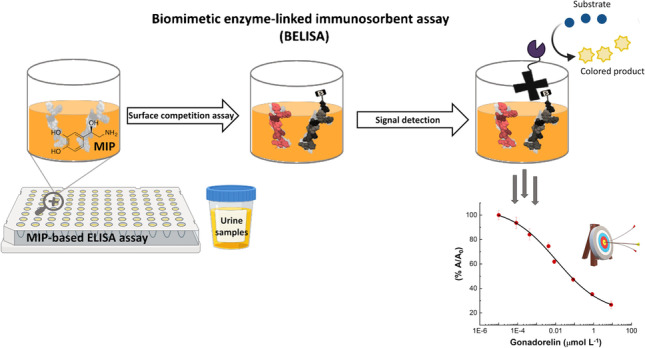
Biomimetic enzyme-linked immunosorbent assay (BELISA)

**Supplementary Information:**

The online version contains supplementary material available at 10.1007/s00216-021-03867-7.

## Introduction

Enzyme-linked immunosorbent assay (ELISA) technology, which conventionally relies on antibody-antigen affinity reaction, is considered one of the most widespread diagnostic tools in clinical routine measurements. It is used to detect and measure a wide variety of analytes belonging to peptides, proteins, viruses, cells, and other organic as well as inorganic molecules of interest in several bioanalytical and biomedical research areas. Besides, it allows the multiplexed screening of different biological samples (e.g., blood, urine, saliva, etc.) on disposable plastic microplates, offering many practical advantages. Moreover, it does not require expensive instrumentation and it is often readily available as commercial kits where the analytical signal is generally based on an optically active product [1]. ELISA has undergone numerous welcomed changes from the first one described by Engvall and Perlmann in 1971 [2]. As a result, the term ELISA now refers to a wide range of micro-welled plate assays, some of which do not involve enzymatic reactions [3–5] and/or immune complex formation [6, 7]. Since current research interest is dictated by the requirement of greater throughput and sensitivity alternatives, as well as cheaper, conventional immunoassays could gradually be replaced by abiotic ELISAs. In this context, several efforts have been already devoted to discovering new signal reporters to increase ELISA sensitivity [3] and to pinpoint capturing agents, i.e., mimetics [8], able to flank and/or supplant antibodies that generally are expensive and sensitive to environmental conditions. Looking at mimetic capturing agents, the literature has highlighted aptamers and molecularly imprinted polymers (MIPs) as the most promising ones to develop diagnostic “antibody-free” ELISA-like format assays, i.e., enzyme-linked oligonucleotide assay (ELONA) [6, 9], biomimetic enzyme–linked immunoassay (BELISA) [10–19], pseudo-ELISA [7, 20–23], or nanoparticle-based assay (MINA) [1, 15, 24–29]. At present, a positive imbalance of the literature is in favor of aptamers, with only a few reports about MIPs starting from 2014. In the latter scenario, the relentless research for new materials used for MIP production, during the last decade, has pushed in the direction of green bio-inspired polymers which not only challenged the classical materials but also contributed to extending the range of their applicability. Among these, the role of “class leading” material must undoubtedly be attributed to polydopamine (PDA), belonging to the class of poly-catecholamines [30–33]. Thanks to the strong self-adhesive properties of PDA, the polymerization can be realized to almost any surface, significantly expanding the applicability of this polymer to a plethora of material supports for (bio)analytical purposes, i.e., metals, semi-metals, glass, and silicon for biosensing; soft (e.g., polydimethylsiloxane, PDMS) and hard polymers used for disposable UV-Vis and fluorescent detection, included ELISA plates; cellulose paper for lateral flow assays (LFA) [34]; and more. Due to the aforementioned and further extremely advantageous features, our research group started working with PDA and developed several smart optical-based assays [35–38] and MIP-based mimetic receptors for biosensing [39]. More recently, also polynorepinephrine (PNE), a close analog of PDA, has displayed improved analytical performances when exploited for MIP synthesis, giving even better results than PDA [40–42]. Catecholamine-based MIPs, in particular PDA and PNE, are very attractive since their polymerization (the mechanistic processes are currently not completely known [43–47]) is obtained spontaneously and under mild aqueous conditions starting from the corresponding low-cost monomers (dopamine (DA) and norepinephrine (NE), respectively), and can be performed in any laboratory as they do not require specific technology (being easy to operate) or expensive devices. In this context, we successfully developed an original PNE-based MIP as capturing receptor for gonadorelin detection both in buffer and synthetic urine [40]. It was coupled to surface plasmon resonance (SPR) transduction and optimized to set up a sensitive and selective “two-step” competitive assay for anti-doping controls [40]. The small decapeptide hormone analyzed, gonadorelin (G), or gonadotropin-releasing hormone (GnRH), is produced by the hypothalamus which activates gonadal function by promoting gonadotropin secretion (FSH and LH) from the anterior pituitary. After reacting with specific receptors located in the pituitary gland, GnRH is internalized and partially destroyed. Therefore, it is not usually found in biological fluids such as plasma and urine. GnRH plays a key role in normal reproduction and in a diverse array of pathophysiological states. In this scenario, several synthetic GnRH agonists (leuprolide, buserelin, nafarelin, deslorelin, etc.) have been developed for therapeutic use to treat different reproductive disorders and hormone-sensitive cancers (breast, pancreatic, and ovarian cancers) etc. The reverse side could be the use of gonadorelin and its analogs for doping purposes in sports since its pulsatile action results in an increased testosterone level with possible improvement in athletic performance. Thus, all these pharmacological drugs are banned by the World Anti-Doping Agency (WADA).

Here, we report and demonstrate the versatility of the developed PNE-based MIP for G, moving from a benchtop instrumental asset (i.e., SPR) toward a portable, low-cost, and very widely used bioanalytical platform, i.e., ELISA reader. To this aim, the MIP for G has been adapted to play the role of the capturing antibody in 96-well microplates, obtaining a very sensitive and selective biomimetic enzyme-linked immunoassay (BELISA) test in a competitive format [34]. This assay is similar to the conventional ELISA, but the traditional bioreceptor, i.e. the antibody, is replaced with a “*household*” PNE-mimetic receptor, showing several advantages in terms of ease and speed of preparation, low cost, temperature stability, and versatility. The whole BELISA was designed to maintain the compatibility with one of the most widely used readouts that involve horseradish peroxidase (HRP) enzyme as a signal reporter and TMB as a substrate that, in its classical use, develops the well-known optical signal in the visible range (*λ*_max_ = 450 nm). Thus, the colorimetric signal obtained was directly related to the amount of G and it was measured by a simple absorbance reader, like those commonly present in clinical laboratories.

## Experimental section

### Materials and chemicals

Gonadorelin European Pharmacopoeia (EP) Reference Standard (acronym G) {pGLU}HWSYGLRPG-NH_2_ (MW = 1182.33 Da), l-norepinephrine hydrochloride (NE, ≧ 98.0%), l-lysine (≧ 98.0%) tris(hydroxymethyl)aminomethane hydrochloride (Tris-HCl, ≧ 99%), l-cysteine hydrochloride monohydrate (≧ 98.%), 4-(2-hydroxyethyl)piperazine-1-ethanesulfonic acid sodium salt (HEPES), sodium chloride, hydrochloric acid, acetic acid (≧ 99.7%), polyoxyethylene sorbitan monooleate (Tween-20), bovine serum albumin (BSA), streptavidin-horseradish peroxidase conjugate (S-HRP), methanol LC-MS grade (99.8%), water LC-MS grade, acetonitrile (ACN), ammonium hydroxide (NH_4_OH), and formic acid (FA ≧ 95%) were all obtained from Merck (Milan, Italy). 3,3′,5,5′-Tetramethylbenzidine (TMB) substrate and stop solution were from Thermo Fisher Scientific (Waltham, MA, USA).

Biotinylated gonadorelin (acronym BG) {pGLU}HWSYGLRPG{Lys(biotin)} (MW = 1537.75 Da) and non-specific control peptides: PEP_A Ac-ISLAPKAQIK-NH_2_ (MW = 1109.37 Da) and PEP_B {pGLU}HWSY{d-LEU}LRP (MW = 1182.34 Da) were synthesized by GenScript (Leiden, Netherlands). Dilution buffer (DB) (10 mmol L^−1^ HEPES, 150 mmol L^−1^ NaCl, pH 7.4) and washing buffer (WB) (10 mmol L^−1^ HEPES, 150 mmol L^−1^ NaCl, 0.0001% BSA, and 0.1% Tween-20, pH 7.4) were used for peptides’ dilutions and wells’ washing, respectively. All buffer solutions were prepared using ultrapure Milli-Q^TM^ water (18.2 MΩ • cm) and were filtered through a microporous filter (Millipore, pore size of 0.22 μm). All chemicals used were of analytical grade. 96-well flat-bottom microtest plates and acetate foil for 96-well plates were obtained by Sarstedt (Nümbrecht, Germany). OASIS cartridges (hydrophilic-lipophilic-balanced [HLB], 60 mg, 3 cm^3^) were acquired from Waters (Milford, MA, USA).

The internal standard (ISTD) (Des-Pyr^1^)-GnRH (MW = 1071.21 Da), used in mass spectrometry analysis, was obtained from Bachem (Bubendorf, Switzerland).

### Methods and instrumentation

#### Preparation of imprinted polymers on microplates

MIPs were directly grown (via a bulk polymerization) onto disposable 96-well microplates by dropping a fresh polymerization mixture (100 μL/well) composed of the functional monomer NE (2.00 g L^−1^ in 10 mmol L^−1^ Tris-HCl, pH 8.50) in the co-presence of the template G (4.23 nmol L^−1^). The plates were left upside down for 5 h at a fixed temperature (*T* = 25.0 ± 0.5 °C), under static conditions, to obtain the polymeric film formation both on the base and walls of each microplate well. Then a surface passivation step was performed to minimize possible non-specific binding of biomolecules, mainly present in biological fluids, such as human urine or serum, to the polymeric surface. An aqueous solution containing 10 mM cysteine (Cys), 10 mM lysine (Lys), and 10 mM tris(hydroxymethyl)aminomethane (Tris) was dropped onto the PNE surface (200 μL/well) and left overnight at 25 °C by avoiding evaporation. This passivation step was allowed by Michael addition reactions through the amines and thiol contained in the used compounds (Cys, Lys, Tris). Washing steps with acetic acid (5% v/v, 200 μL/well for 3 times) and deionized water (200 μL/well for 3 times) were accomplished to remove the template from the polymeric binding cavities and any residual oligomers. Between washes, the plates were inverted and blotted against absorbent paper.

#### Competitive assay for gonadorelin analysis

**Scheme 1:**
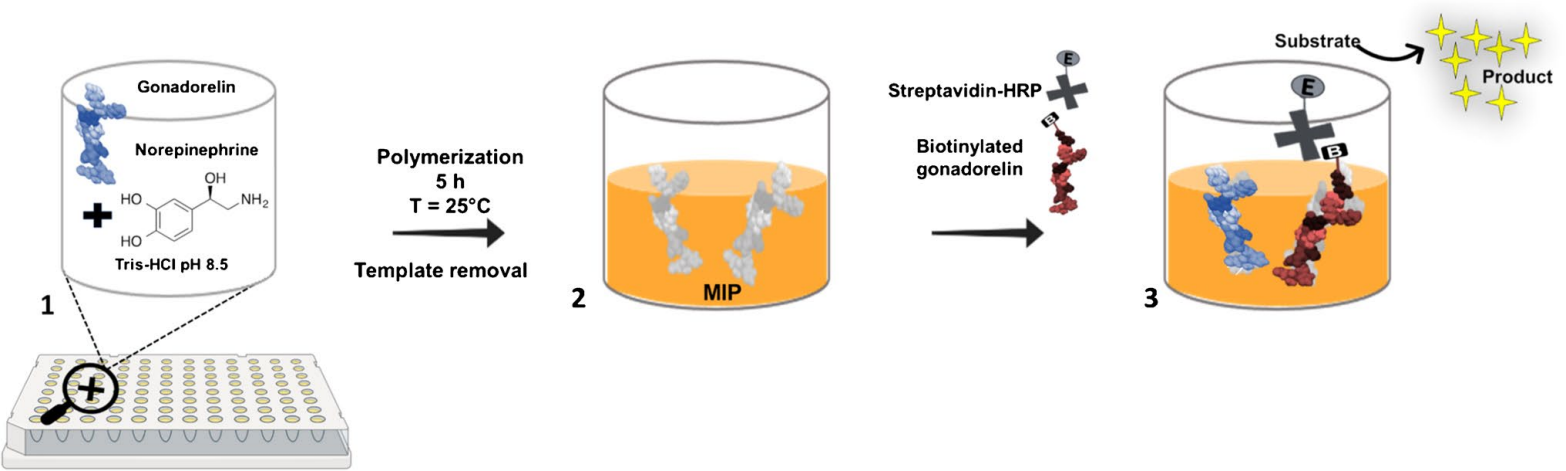
Sketched illustration of the imprinting process onto a micro-welled plate. (1) The functional monomer (norepinephrine) and the template (gonadorelin) were dropped as a mixture onto the wells’ microplate, and the polymerization process occurred for 5 h at 25 °C. (2) Then, the template was washed out the polymeric matrix, and the “two-steps” competitive BELISA (3) was set up

The competitive BELISA for the quantitative detection of G in standard solutions and human urine was designed by employing a two-step assay architecture over the MIP (see Scheme 1). The first part of the study involved the preliminary assessment of the BG binding affinity to the synthesized MIP (see paragraph *a* below). Afterward, the competition was established by sequential incubations of the analyte G in water and its competitor BG conjugated with streptavidin-HRP (see paragraph *b* below), respectively. The last step involved the enzyme S-HRP cascade process to obtain a colorimetric readout signal. In detail, the S-HRP optimization was carried out using two blocking solutions, 0.0001% BSA and 0.1% Tween-20, contained in the WB which contribute to minimize possible non-specific binding by avoiding any masking of the MIP cavities. The blocking reagents, 0.0001% BSA and 0.1% Tween-20, used in this bioassay were estimated using the approach proposed by Steinitz [48] as a compromise between high specific signal and low non-specific background signal.

- *Biotinylated gonadorelin calibration*

The protocol for calibrating BG is listed below: the MIP-coated wells were first conditioned by washing with WB (200 μL/well) followed by incubating BG at different concentrations (0.20–13.00 μmol L^−1^ diluted in DB). BG solutions were dispensed in triplicate into the modified wells. The microplate was gently stirred for 10 min and incubated at 4 °C for 40 min. A washing step with WB (200 μL/well) was then performed, followed by streptavidin-HRP enzyme (0.20 mg L^−1^) incubation (detecting molecule S-HRP, 200 μL/well) for 30 min at 4 °C. The wells were finally washed with WB (200 μL/well for three times), and TMB reagent (100 μL/well) was added and incubated for 5 min before the enzymatic reaction was stopped by the addition of 0.16 M H_2_SO_4_ (stop solution, 100 μL/well). The absorbance was measured by using iMark^TM^ microplate absorbance reader (Bio-Rad, Milan, Italy) at *λ*_max_ = 450 nm.

b) *Competitive inhibition MIP-based assay*

The PNE-based MIP was used in a competitive BELISA to quantify G through a competition between a different concentration of free gonadorelin and a fixed amount of BG.

The free analyte G diluted in DB, in the concentration range 8.46•10^−5^–8.46 μmol L^−1^, was dispensed into each well (100 μL/well) of the microplate. The solution was blended, incubated at 4 °C for 20 min, and then washed with WB (200 μL/well). Subsequently, the procedure continues as described in the paragraph above by exploiting a fixed concentration of BG (6.50 μmol L^−1^, 100 μL/well). Besides, the MIP selectivity was assessed in the competitive BELISA by measuring the response of unrelated peptides (PEP_A and PEP_B; see details in “Materials and chemicals”) which replace the analyte G incubation.

#### Analysis of gonadorelin in human urine samples

The two-step competitive assay was tested on human urines from healthy volunteers (see ethical standard).

Samples were chosen among those intended for disposal at the Clinical Pathology Laboratory of the University Hospital in Pisa. Detailing, samples were selected among those that did not present significant amounts of proteins, as verified by nephelometric measurements, and which contained negligible G concentration (see mass spectrometric analysis of unspiked urine). Hence, the urine samples were diluted 1:10 in water and spiked with a known amount of gonadorelin spanning from 0.084 to 846 nmol L^−1^ to simulate post-administered human urine specimens. Spiked and unspiked samples were explored following the same experimental procedure, except for G addition, to evaluate the possible occurrence of matrix effects.

#### Urine samples analysis by LC-MS/MS

Mass spectrometry was used as an analytical gold standard technique for G quantification to validate the designed competitive BELISA. To this aim, blank and G-spiked urine samples from healthy volunteers were analyzed by LC-MS/MS and BELISA test in parallel. For LC-MS/MS, the samples were diluted 1:10 in a water/methanol/formic acid (97:2.9:0.1; v/v/v) solution and spiked with G at different concentrations (0–0.084–0.423 and 4.23 nmol L^−1^). The ISTD (Des-Pyr^1^)-GnRH was added to these fortified urine samples to obtain a fixed concentration of 100 ng mL^−1^ and then extracted and purified via solid-phase extraction (SPE) using Waters Oasis-HLB^TM^ cartridges (3 mL, 60 mg), as previously reported by Zvereva et al. [49]. Before the sample elution, the cartridges were preconditioned by flowing methanol (2 mL) and water (2 mL) and by rinsing with NH_4_OH/H_2_O (5:95 v/v, 2 mL) and with ACN/H_2_O (20:80 v/v, 2 mL). The sample elution was performed with 5% FA (2 mL) in an ACN/H_2_O (75:25 v/v) solution, and the eluates, thermostated at 45 °C, were evaporated to dryness under a nitrogen stream and reconstituted with the diluent solution. Referring to Thomas et al. [50], an implemented mass spectrometry method was here developed. Sample analysis was performed on an LC-MS/MS instrumental layout composed of an Agilent (Santa Clara, CA, USA) 1290 UHPLC system which consists of a binary pump, a column oven set to 60 °C, and a thermostated autosampler. This is coupled with an AB Sciex (Concord, Ontario, Canada) QTRAP 6500+ mass spectrometer working as a triple quadrupole and equipped with an IonDrive™ Turbo V source. Chromatographic separation was achieved by using an Agilent Zorbax StableBond 300 C18, 1 × 50-mm, 3.5-μm column, while the integrated switching valve was used to discard both head and tail of the HPLC runs.

The mobile phases were constituted of (A) 0.1% FA in methanol and (B) 0.1% formic acid in water and the gradient elution (100 μL/min flow rate) was performed as follows: 0.0 min (A) 5%, 5.2 min (A) 50%, 6.2–7.2 min (A) 90%, 7.7–10.5 (A) 5% (volume injection = 5 μL). A mass spectrometry selected reaction monitoring (SRM) method was operated in positive ion mode. For each compound, after the optimization of the declustering potential (DP), collision energy (CE), and collision exit potential (CXP), three transitions were considered in the analysis. Based on the highest signal/noise ratios, one of them was used as quantifier (Q) and the others as qualifiers (q) (see ESM, Table S1). Additional operative parameters were set as follows: collision gas (CAD) nitrogen; operative pression with CAD gas ON, 3.6 mPa; curtain gas (CUR) = 20 arbitrary units; gas source 1 (GS1) = 40 arbitrary units; gas source 2 (GS2) = 45 arbitrary units; ion spray voltage (ISV) = 5.5 kV; source temperature (TEM) = 550 °C; entrance potential (EP) = 10.3 V; and DP = 70 V.

## Results and discussion

In this study, a PNE-based mimetic receptor to capture gonadorelin (G) was produced, as a first attempt, onto disposable miniaturized 96-well microplates (see Scheme 1) for easier and cost-effective detection of poorly or non-immunogenic small molecules, here G. The binding capacity, as well as synthesis conditions, of the MIP toward G was previously characterized through a benchtop sensing platform (surface plasmon resonance—SPR) by growing the polymer layer onto a planar gold support [40]. The MIP showed very interesting analytical behavior, overall, in terms of *K*_D_ and LOD. Thus, the opportunity to adapt the PNE-based mimetic assay to an ELISA-like test arose. For this purpose, the synthesis conditions and dilution buffer (DB) were slightly modified according to the previous study [40] (as detailed in the “Experimental” section) to be tailored to the different support structure, here polystyrene microplates, and assay mode (static conditions instead of continuous flow in SPR). To the best of our knowledge, up to this point, there are only a few competitive ELISA tests based on antibodies for G detection, available on the market as a research tool, which could be tentatively applied in the anti-doping field. Nevertheless, the unknown antibody-binding epitope and the scarce information about the immunogen used to elicit the immune response limit their applicability. The colorimetric two-step competitive assay to quantify the analyte G was directly carried out on the MIP surface. For this purpose, a fixed concentration of the competitor molecule, the biotinylated G (BG) conjugated to the signal reporter (S-HRP), was involved in the assay to trigger a competitive reaction with G for the same MIP cavities. The assay exploits one of the most common ELISA readout strategies, i.e.*,* the use of horseradish peroxidase (HRP) as a signal reporter, and tetramethylbenzidine (TMB) as substrate, developing a signal in the visible range (*λ*_max_ = 450 nm).

The BELISA development has required the optimization of several parameters, from the buffer composition for the washing steps (WB) and binding interaction with the MIP (DB) to the optimal concentration of the BG competitor, as well as the concentration of the detecting molecule S-HRP. The working temperature and the incubation times for the different steps (MIP-analyte, -BG, and BG-S-HRP interactions) were tried out to optimize the assay. Finally, the developed BELISA was calibrated and applied to human urine fortified with G. Mass spectrometric analysis was separately settled and eventually performed to validate the BELISA test (see paragraph below).

### Evaluation of factors affecting the bioassay and BG binding test

**Fig. 1:**
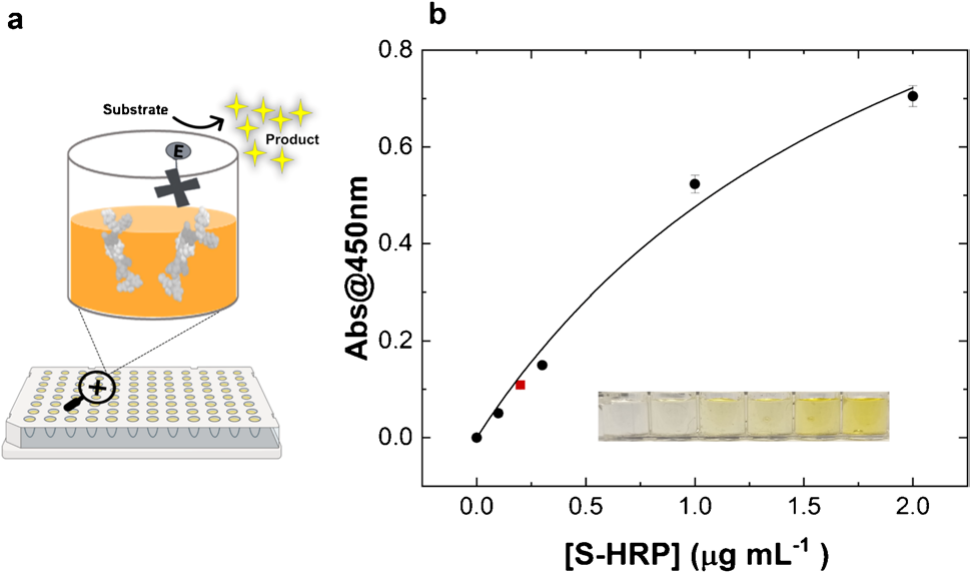
**a** Schematic depiction of the assay setup to select the S-HRP concentration (0.20 μg mL^−1^); **b** calibration curve of the enzyme conjugated to streptavidin (S-HRP) on the MIP-coated microplate

To exclude possible optical artifacts due to the sole presence of S-HRP, TMB, and/or the reaction cascade involved in the readout step on the MIP, a dose-response test was first carried out. To this aim, various S-HRP enzyme concentrations in WB were dispensed into the MIP-coated wells and kept at 4 °C for 30 min, followed by three washing steps with the same buffer (200 μL/well). Figure 1 displays the curve obtained after S-HRP addition at increasing concentration. Accordingly, we selected the optimal S-HRP concentration giving the lowest Abs@450 nm, which significantly differs from the blank (no S-HRP added into the wells), resulting in 0.20 μg mL^−1^ (dilution ratio 1:5000 in WB). This concentration was thus employed for all further experiments. This first step demonstrated that the polymer does not interfere with the enzyme-based optical detection and is the prerequisite to go further in the assay development.

Thereafter, the BG calibration, the competitor molecule, was tested and optimized. Two temperatures, 4 °C and 25 °C, were compared in terms of BG binding efficacy on the PNE-based MIP (see ESM, Fig. S1). These temperatures were selected since they are most used in standard ELISA protocols for reagents’ incubation. Regarding this, we aimed to simplify the conventional ELISA kit, by substituting the natural receptors with biomimetics, while minimizing the standard ELISA protocol in terms of incubation temperatures, operative steps, and laboratory equipment required. Besides, these temperatures are also chiefly used for the storage of substrates modified with natural inspired polymers (e.g., PNE and PDA). The calibration curves were obtained by plotting the Abs@450 nm against the concentrations tested both at *T* = 4 °C (solid line) and *T* = 25 °C (dashed line), as displayed in Fig. S1 (see ESM). In this case, the test performed at *T* = 25 °C provides a saturation level higher (around 13.00 μmol L^−1^) than the curve resulting from a 4 °C incubation experiment (around 6.50 μmol L^−1^). Nonetheless, these data (*T* = 25 °C) are affected by a higher standard deviation. According to the latter evidence, the overall assay reproducibility at 4 °C resulted slightly improved, as inferred by the intra-assay average coefficient of variation (% coefficient of variation = 100* standard deviation/mean), _av_CV% (25 °C) = 4.40% and _av_CV% (4 °C) = 3.44%, respectively. This, in addition to being well compatible with routine ELISA incubation temperature, allows avoiding local temperature fluctuations over seasons and among different laboratories, improving the assay repeatability. This bestows robustness to the assay as also denoted by the good inter-laboratory reproducibility which was estimated at _av_CV%_4°C_ = 4.8% at 4 °C.

Lastly, the equilibrium dissociation constants, *K*_D_, were extrapolated from the saturation curves obtained by measuring the direct BG-MIP binding interaction. In this case, the two datasets (see ESM, Fig. S1) were fitted with a Langmuir binding model (1:1) by the equation *y* = (*R*_Absmax_ * *X*)/(*K*_D_ + X) (Origin 2019b), where *R*_Absmax_ is the maximum response (Abs@450nm) and *K*_D_ is the equilibrium dissociation constant that was estimated by the two curves. From this evaluation, *R*_max_ and *K*_D_ values resulted as follows: *R*_max_ (25 °C) = 0.440 ± 0.023, *K*_D_ (25 °C) = 3.03 ± 0.42 μmol L^−1^ and *R*_max_ (4 °C) = 0.317 ± 0.015, *K*_D_ (4 °C) = 1.24 ± 0.20 μmol L^−1^.

**Fig. 2:**
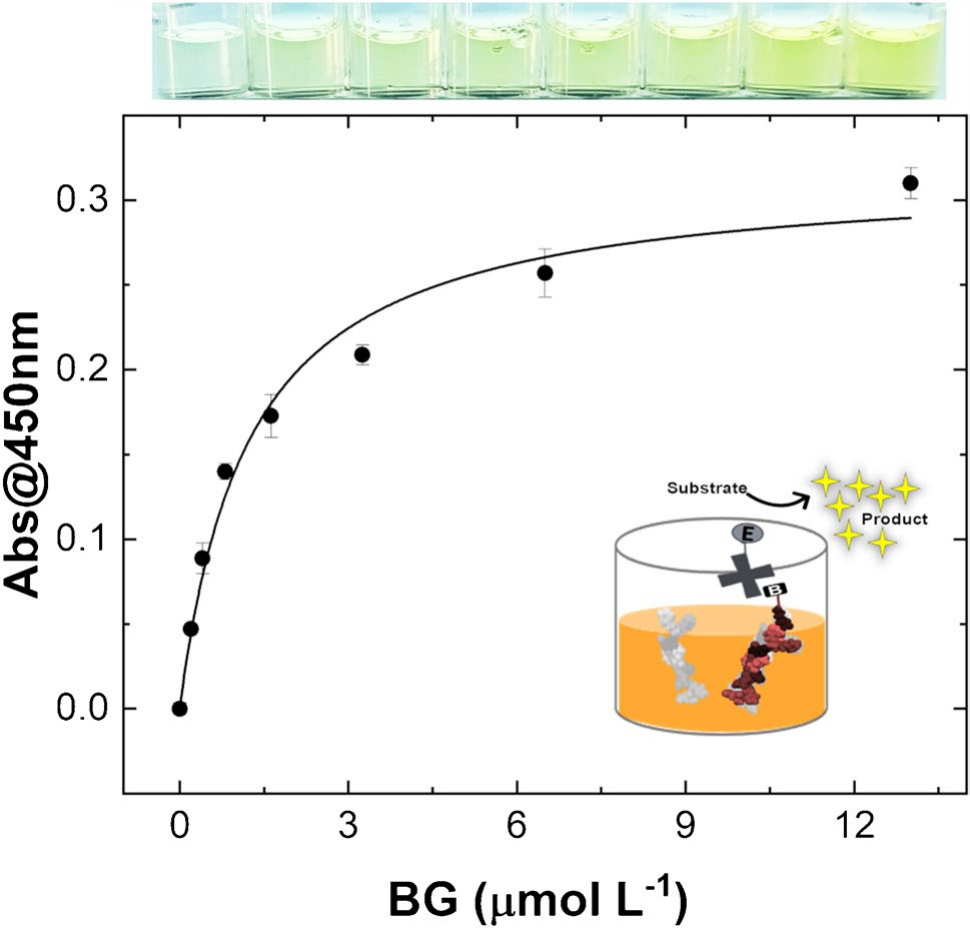
Saturation binding curve for BG where the BG concentration (0.20–13.00 μmol L^−1^) was plotted against Abs@450nm. Each point indicates the mean response ± SD of quadruplicate measures. The inset figure reports the BG calibration scheme. An illustrative photo (at the top of the figure) reports the wells’ color development for an increasing series of BG concentrations (from left to right)

Finally, for achieving an efficient competition, the optimal competitor molecule (BG) concentration has been selected after its calibration. In this case, the BG concentration was derived from the *K*_D_ estimation at 4 °C (see Fig. 2) and experimentally selected at 6.50 μmol L^−1^. Notably, the estimated *K*_D_ value of the interaction MIP-BG extrapolated from the BELISA is in the same order of magnitude as the one previously inferred by the SPR biosensor [40]. This finding is very interesting since the surface binding sites (surface capacity) of the two assay substrates (polystyrene microwell vs gold chip) are very different in size and material, encouraging further applications of PNE-based mimetics on both platforms.

### Competitive binding assay on MIP-coated microplates

**Fig. 3:**
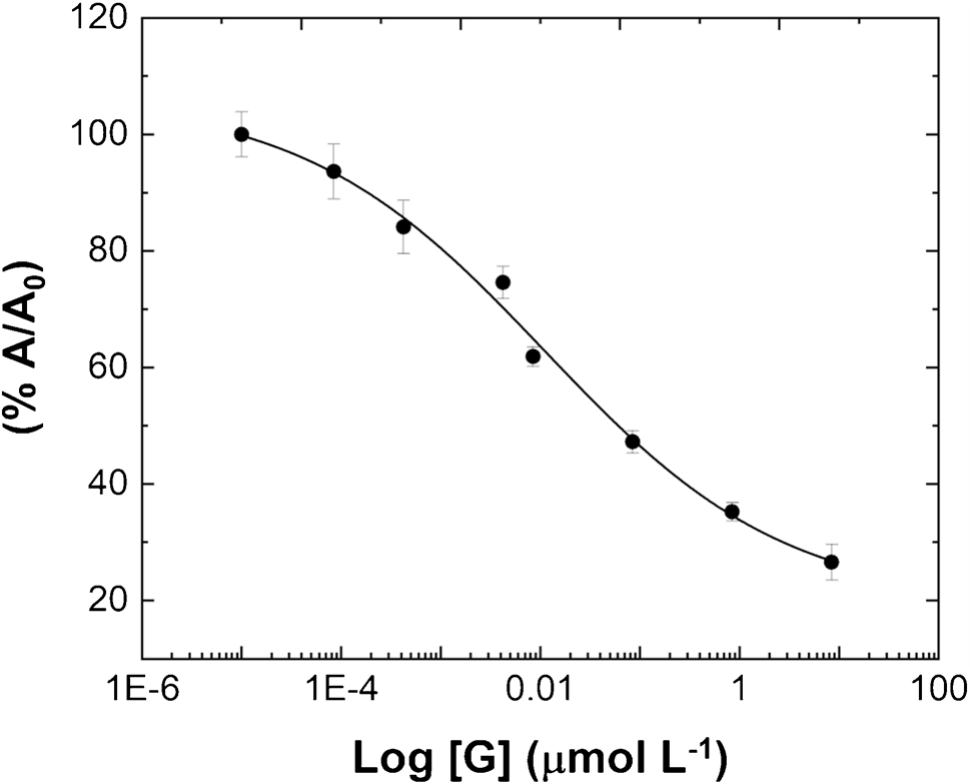
MIP-based competitive inhibition BELISA: typical S-shaped curve (*x*-axis logarithmically transformed) for gonadorelin spiked in standard solutions over the concentration range of 8.46•10^−5^–8.46 μmol L^−1^. The curve was fitted by using a 4-parameter (4PL) logistic regression

Once the main experimental conditions were optimized, we moved forward with the actual “two-step” competitive assay by calibrating gonadorelin in buffer condition first and, finally, in a real matrix, i.e., human urine. In the first step, the analyte is incubated in the MIP-coated microplate wells (as detailed in the “Methods and instrumentation” section (b)); thereafter, a fixed amount of BG is sequentially incubated at the defined optimal concentration, followed by a washing step and a standard HRP/TMB-based readout protocol. The standard curve obtained in buffer (Fig. 3) within the range 8.46•10^−5^–8.46 μmol L^−1^ showed an evident inhibition of the absorbance signals with the increase of G concentration, demonstrating the ability of G to compete with BG. This is a key result obtained by successfully tuning the BG concentration to be used in the assay, as aforementioned. The signals measured (Abs@450nm) were expressed as the percent of analyte G bound to the MIP binding sites (*A*) divided by the maximal binding of the competitor molecule BG (*A*_0_) to the MIP surface, measured in absence of the analyte (blank) (%*A*/*A*_0_). In detail, data are reported in Fig. 3 as a semilogarithmic plot to display the typical S-shaped sigmoidal curve of the competitive BELISA. The curve is fitted with a 4-parameter logistic (4PL) regression (*R*^2^ = 0.989) by the equation $y=\frac{\left({A}_1-{A}_2\right)}{1+{\left(x/{x}_0\right)}^p}+{A}_2$ where *A*_1_ is the theoretical response at zero G concentration (*A*_1_ = 106.3 ± 6.5 μmol L^−1^) and *A*_2_ the theoretical response at infinite concentration (*A*_1_ = 19.4 ± 7.1 μmol L^−1^); *p* is the slope factor (*p* = 0.36 ± 0.09 μmol L^−1^ ), and *x*_0_ is the mid-range concentration (inflection point, *x*_0_ = 0.011 ± 0.005 μmol L^−1^ ). The sigmoidal curve showed a linear response in the concentration range spanning from 0.42 to 846 nmol L^−1^ (see ESM, Fig. S2). The limits of detection (LOD) and quantification (LOQ) were calculated as three times and ten times, respectively, of the standard deviation for the blank samples interpolated in the sigmoidal curve, namely LOD = 277 pmol L^−1^ and LOQ = 14.1 nmol L^−1^. In addition, in terms of reproducibility, the BELISA performed very well, with _av_CV% = 4.07.

### Selectivity of the BELISA for gonadorelin

**Fig. 4:**
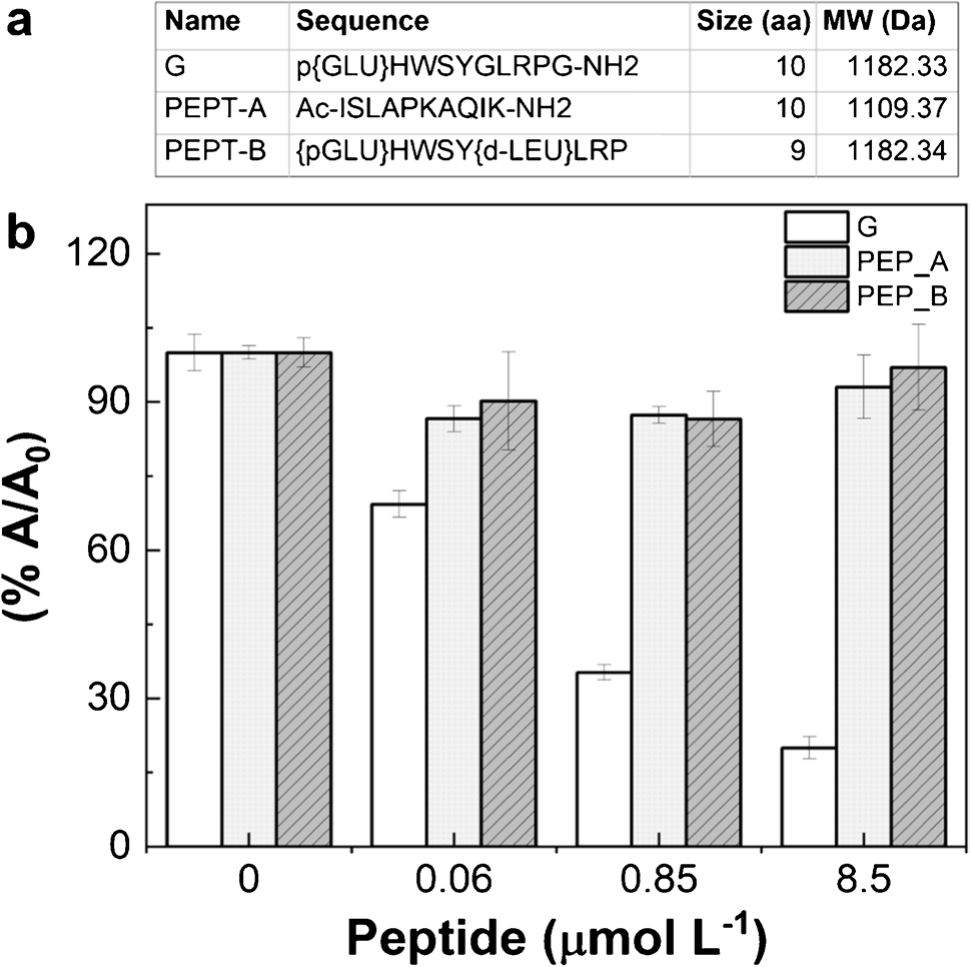
Selectivity test performed on MIP-coated microplates. **a** Sequence, number of amino acids, and molecular weight of each peptide tested; **b** the competitive assay was performed with the target G (white color) and unrelated control peptides (PEP_A, gray bars, and PEP_B, gray striped bars)

The selectivity of the assay toward the analyte G was assessed by measuring the BELISA response (Abs@450nm) to two different control non-related peptides with comparable molecular weight and number of amino acids to the analyte G (see table in Fig. 4a). To this, the two peptides were added to the MIP surface as the first step in place of the analyte. Compared to G behavior (Fig. 4), almost negligible inhibition responses (less than 10%, expressed as *A*/*A*_0_ * 100) were found for both the unrelated peptides A and B. This means that the BG competitor may bind the MIP at the same level in all the cases and very low or no competition with the nonspecific peptides occurs. Therefore, non-related peptides compete very poorly with BG for the same MIP cavities, confirming the very good selectivity of the assay for the imprinted target. This also demonstrates that the PNE-based mimetic retains this feature also in batch (static) conditions, consolidating data previously obtained on the SPR biosensor [40].

### Analysis of gonadorelin in human urine by BELISA

**Fig. 5:**
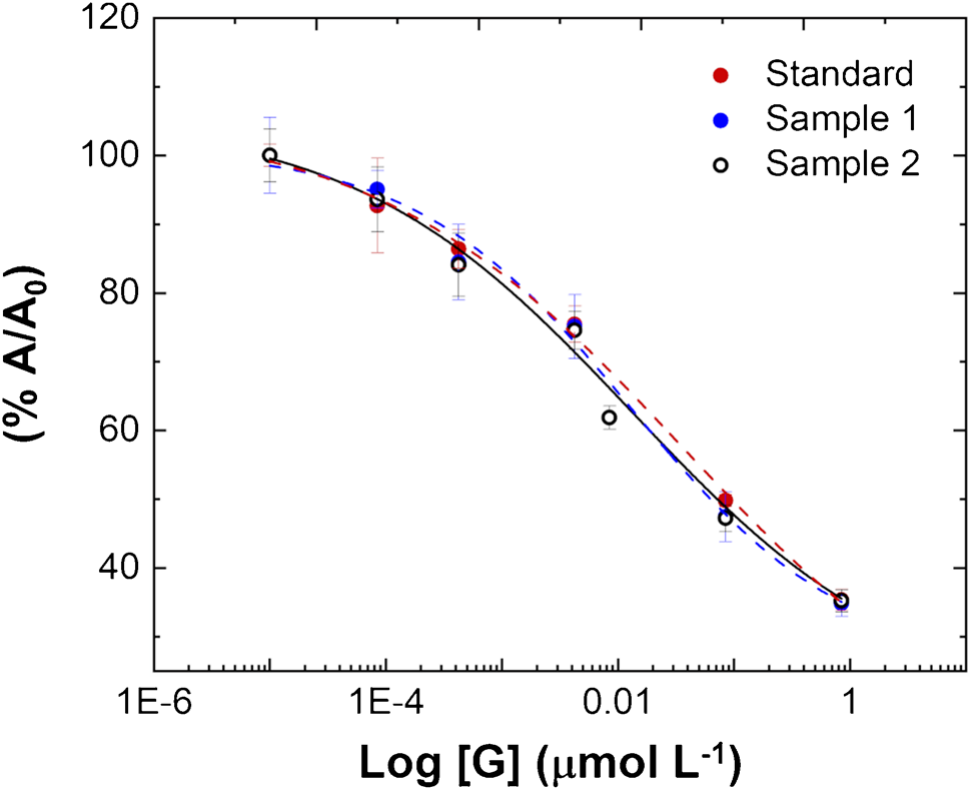
(a) Competitive inhibition curve obtained by performing the MIP-based BELISA on human urine samples and standard solution fortified with gonadorelin, spanning from 0.084 to 846 nmol L^−1^

To evaluate the applicability of the novel competitive MIP-based BELISA, human urine samples from two healthy volunteers were tested. Preliminary experiments were conducted on untreated urine since the direct analysis of samples without the need of pre-analytical steps is one of the most important requirements in ELISA-like assays. However, the “as it is” urine testing resulted in no signal development possibly due to some urine component that impaired the test function. The stumbling block has been overcome with a 1:10 dilution of the urine fortified with G (0.084–846 nmol L^−1^ in aqueous solution), significantly improving the output signal. The diluted urine samples were thus dispensed into the microplate, and then the assay procedure proceeded as described above (as detailed in the “Methods and instrumentation” section (b)). In this case, a successful competition occurred (Fig. 5), and the concentration of G in urine specimens was determined using the calibration obtained in buffer (Fig. 3), performed on the same day, to estimate the mean recovery. The mean recovery, expressing the closeness of the concentration values obtained in biological samples, with respect to the nominal spiked concentration, was calculated by the following formula _av_recovery% = % (measured concentration − basal concentration/added concentration [51]). The basal G concentration in urine specimens is almost zero (see the following LC-MS/MS analysis); thus, the resulting _av_recovery% was equal to 91 ± 19% for urine sample 1 and 96 ± 25% for urine sample 2. The values obtained indicate a good recovery albeit a recovery overestimation occurred at the lowest concentration tested (recovery% = 118% for sample 1 and 124% for sample 2). Moreover, the reproducibility on real samples resulted very good, namely _av_CV% = 3.49 for sample 1 and _av_CV% = 5.24 for sample 2. Lastly, the BELISA was validated by mass spectrometry which is the reference analytical technique required by WADA in anti-doping controls.

### LC-MS/MS method validation

**Fig. 6:**
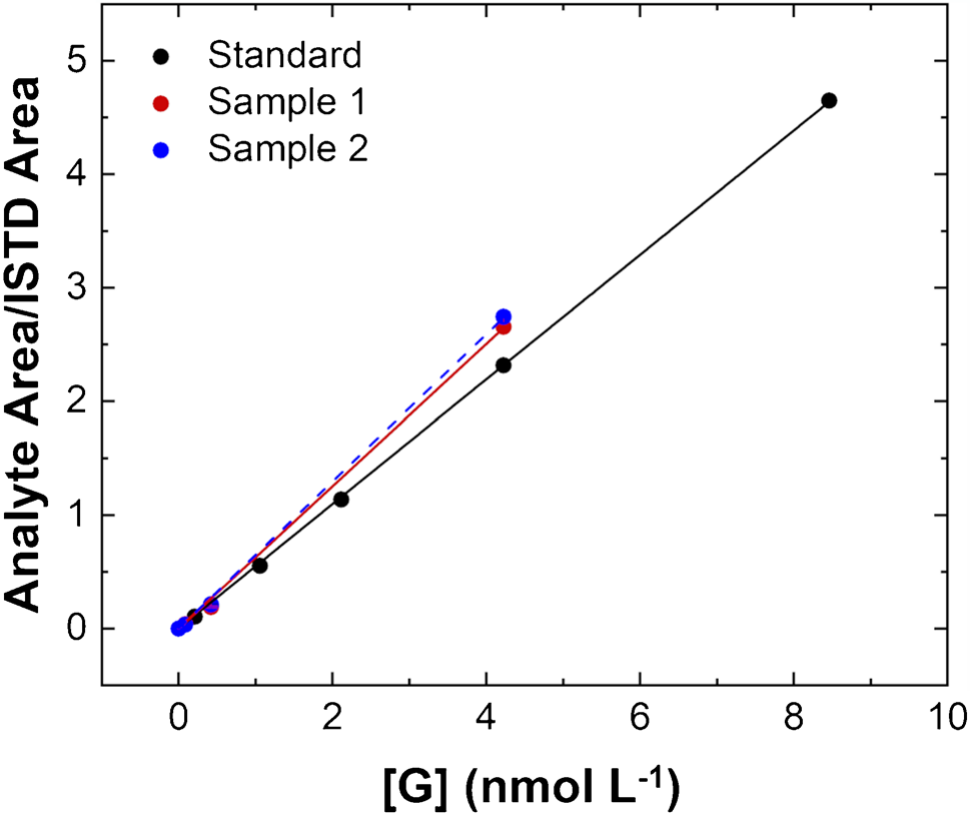
Calibration curve for the LC-MS/MS analysis of gonadorelin (G) in standard solutions (concentration range: 0.084–8.46 nmol L^−1^, black dots) and results (red and blue dots) of urine specimens spiked with G at 0.084, 0.423, and 4.23 nmol L^−1^

LC-MS/MS analysis was set up by modifying a previous protocol reported by Thomas et al. [38]. Human urine samples were in parallel analyzed with BELISA and LC tandem mass spectrometry, in this case in compliance with European Medicines Agency (EMA) guidelines to assure the quantitative performance of the assay (including selectivity, linearity, sensitivity, and accuracy) [52]. Firstly, a calibration curve built with standard solutions of G in water-diluted urine (1:10), in the concentration range spanning from 0.084 to 8.46 nmol L^−1^, was performed by LC-MS/MS (Fig. 6). Then the spiked urine samples were analyzed, and the G concentrations were estimated based on the response of the instrument to the known G standard solutions. All the figures of merit were estimated. Initially, the selectivity of the proposed method was assessed by performing repeated injections of urine samples, with and without analyte G, into the chromatographic system and the retention time (RT) was monitored, RT (G) = 4.58 min and RT (ISTD) = 3.88 min (ESM, Fig. S4). No interferences in the selected transitions occurred, demonstrating the selectivity of the method. The linearity of the method was evaluated for the G calibration curve (*y* = *a* + *bx*, where *a* = − 0.022 ± 0.006 and *b* = 0.551 ± 0.003), carried out in standard solutions, and it was expressed as correlation coefficient (*R*) ≥ 0.999.

The G concentrations in standard solutions providing a “signal-to-noise” (S/N) ratio close to 3 and 10, calculated using a specific tool of ABSciex Analyst® software, were assumed as LOD and LOQ, respectively. The LOD achieved corresponds to 21 pmol L^−1^ while the LOQ is equal to 84 pmol L^−1^. The accuracy (%) was determined in human urine samples diluted 1:10 in water at 0.11, 0.61, and 1.27 nmol L^−1^, and it was calculated by the following formula: % (measured concentration/nominal spiked concentration). Thus, the resulting accuracy was found in the optimal range of 85–115% as indicated by EMA [52]. Besides, recovery (%) and matrix effect were studied as reported by Matuszewski et al. [51]. The mean percent recovery was established by comparing the peak areas of G and those of ISTD which were added respectively before and after the extraction procedure. The obtained recovery (%) for G was equal to 36.25 ± 1.05 while the recovery (%) for ISDT corresponded to 60.1 ± 0.8. In addition, the matrix effect (% ME) was calculated by comparing the peak areas of the ISTD and those of the analyte G which were respectively added to water (A) and urine (B) samples [%(*B*/*A*)], both previously subjected to extraction procedure (%ME_G_ = 47.4 ± 9.9 and %ME_ISTD_ = 88.1 ± 16.3). The recovery ratio (RR) between the analyte G and the ISTD (Des-pyr^1^)-GnRH was eventually found constant and concentration-independent. Therefore, RR is able to provide a correct analysis and quantification of G in human urine samples.

Additional experiments were executed to study the stability of GnRH and ISTD (Des-pyr1)-GnRH as a result of freeze-thaw cycles. Aliquots of fresh gonadorelin at a low, medium, and high concentration were prepared in water and were divided into two aliquots. The first aliquot was quickly injected into the instrument while the second one was frozen at − 20 °C and thawed at room temperature before being analyzed. The acquired results were compared, and no peptide (G) and/or ISTD degradation was detected. This is an important information confirming the stability of the reference lyophilized peptide reconstituted in water and stored at – 20 °C which was then to be gradually used in the assay’s trials.

### Bland-Altman analysis: evaluation of agreement between BELISA vs LC-MS/MS for measuring gonadorelin

**Fig. 7:**
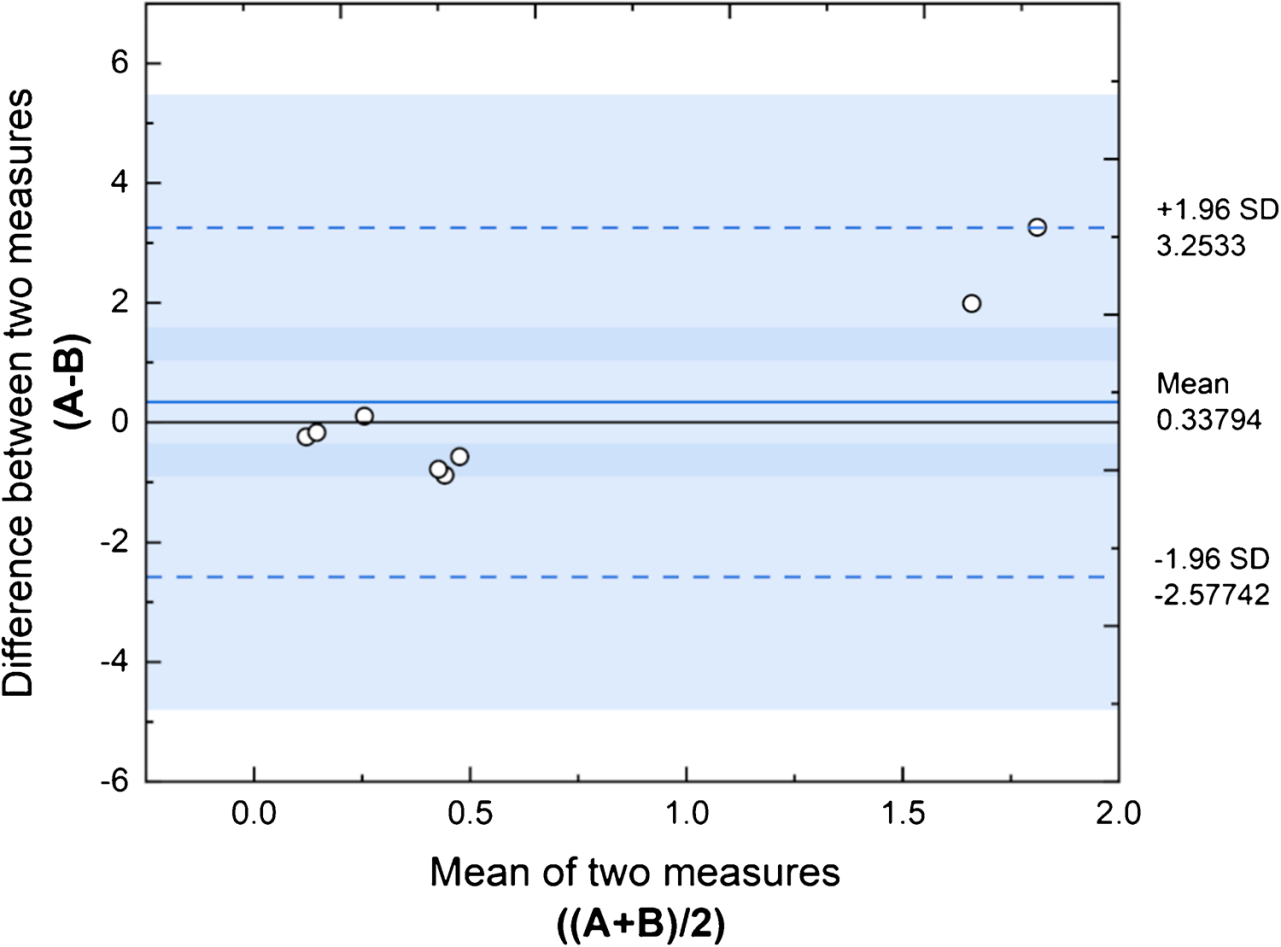
Bland-Altman plot displays the regression line between measurements performed by BELISA (*A*) and the LC-MS/MS reference method (*B*). The difference of each value pair (*A* − *B*) is plotted on the *y*-axis against the mean of each value pair on the *x*-axis ((*A* + *B*)/2)

The correlation between the BELISA test (A) here developed and the reference method by LC-MS/MS (B) was assessed using a Bland-Altman plot (aka Tukey mean-difference plot) [53] (https://www.originlab.com/Bland-Altman-Plots). This is a simple method of data plotting used both analytical chemistry and biomedicine to compare two different analytical strategies. In this case, the agreement between two quantitative measurements was evaluated by constructing a scatter plot, in which the *y*-axis reports the difference between the two measurements (*A* − *B*) while the *x*-axis describes the mean of the value pair ((*A* + *B*)/2). On this basis, the correlation between the paired data reported in Fig. 7 shows that all *xy* scatter points lie within a predetermined interval of ± 2SD of the mean difference (https://www.originlab.com/Bland-Altman-Plots) suggesting that the confidence level between the two methods is equal to 95%. In this case, a good agreement between BELISA and the LC-MS/MS method was achieved.

## Conclusion

In this work, an antibody-free competitive Biomimetic-ELISA (BELISA) based on a nature-inspired molecularly imprinted polymer, as a recognition element, was developed for the successful quantitation of the short peptide gonadorelin. The perspective development of mimetic receptors alternative to antibodies in ELISA-like tests is an extremely exciting and challenging task for the bioanalytic field. This is also in line with the EU Directive (2020/63/EU) on the protection of animals used for scientific purposes and the recent EURL ECVAM recommendations on non-animal-derived antibodies [54].

The aim of the study was the conservation of a routine ELISA-type test protocol coupled to a classical enzyme-based readout at *λ*_max_ = 450 nm, except for the need of antibodies. According to our knowledge, this is the first example of a BELISA test involving a molecularly imprinted polymer based on polynorepinephrine, and able to detect the analyte (gonadorelin, G) in buffer and untreated (except for 1:10 dilution) human urine. The BELISA allowed the sensitive and selective measurement of this potential doping peptide, G, in representative urine samples with very good performance. The results obtained in standards solutions and urine samples showed good reproducibility (_av_CV%=4.07% for standard solutions, avCV% (sample 1) = 3.49%, and avCV% (sample 2) = 5.24%), a challenging task considering that all the receptors were manually drop casted and polymerized in each microplate well. The prospective improvement of this parameter can therefore be figured out by automating, for example, the microwell preparation under highly controlled conditions. To validate the BELISA test, simulated blinded urine samples spiked with G were simultaneously dosed by BELISA and LC/MS-MS, the reference benchtop platform in anti-doping analysis for such type of analytes. In both cases, the detection limit achieved in standard solutions (LOD_BELISA_ = 277 pmol L^−1^ and LOD_LC-MS/MS_ = 21 pmol L^−1^) for G detection resulted in line with the Minimum Required Performance Level (MRPL = 1.69 nmol L^−1^) at which all the World Anti-Doping (WADA)–accredited laboratories must operate. The results from the two platforms correlate well by performing a simple statistical Bland-Altman analysis. The LC-MS/MS method resulted more sensitive than the BELISA, as expected from a gold standard method. Nevertheless, the BELISA may be largely enhanced, if needed, by involving more sensitive detection strategies (e.g., fluorescence or chemiluminescence). As a whole, catecholamine-based MIPs, PNE in this case, have shown (here for the G case study) to be an excellent candidate material to imagine ELISA-like tests of the near future, antibody-free, extremely cheap, able to be prepared for a large variety of targets (even small non-immunogenic), very stable to environmental conditions, and highly versatile. This approach could further open new possibilities as abiotic point-of-care testing to make multiplexing biological sample analysis by integrating the smartphone technology which can be also extendable to several analytes.

## Supplementary Information

ESM 1 (DOCX 20799 kb)

## Declarations

The use of the selected biological samples has been approved by the local Ethics Committee (CEAVNO), Pisa, Italy (19/02/2020).
