# Supplementary material for: A biomimetic enzyme-linked immunosorbent assay (BELISA) for the analysis of gonadorelin by using molecularly imprinted polymer-coated microplates
Source: Anal Bioanal Chem. 2022 Jan 13;414(18):5423–34. doi: 10.1007/s00216-021-03867-7 (PMC9242967; doi:10.1007/s00216-021-03867-7)
Supplement: Supplementary file 1 — (DOCX 20799 kb) [file 216_2021_3867_MOESM1_ESM.docx]

**A biomimetic enzyme-linked immunosorbent assay (BELISA) for the analysis of gonadorelin by using molecularly imprinted polymer-coated microplates**

F. Torrini^1^, L. Caponi^2^, A. Bertolini^3^, P. Palladino^1^, F. Cipolli ^2^, A. Saba^3^, A. Paolicchi^2^, S. Scarano^1^*, M. Minunni^1^*

^1^ Department of Chemistry Ugo Schiff’, University of Florence, Sesto Fiorentino (FI), Italy

^2^ Laboratory of Clinical Pathology, University Hospital of Pisa, Pisa, Italy

^3^ Department of Surgical, Medical and Molecular Pathology and Critical Care Medicine, University of Pisa, Pisa, Italy

*Corresponding author: [simona.scarano@unifi.it](mailto:simona.scarano@unifi.it), [maria.minunni@unifi.it](mailto:maria.minunni@unifi.it)

**Electronic Supplementary Material (ESM)**

**BELISA assay**

**
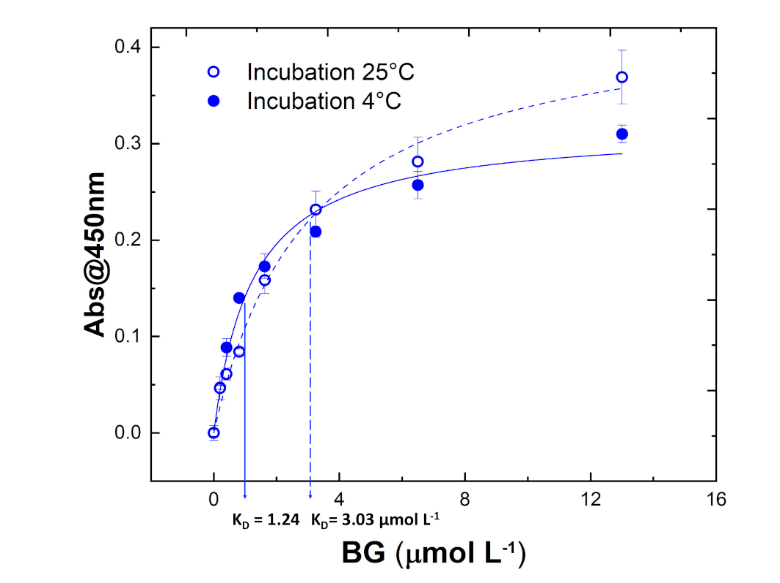
**

**Fig. S1** Comparison of BG calibration curves performed at 4°C (solid line) and 25°C (dashed line), respectively


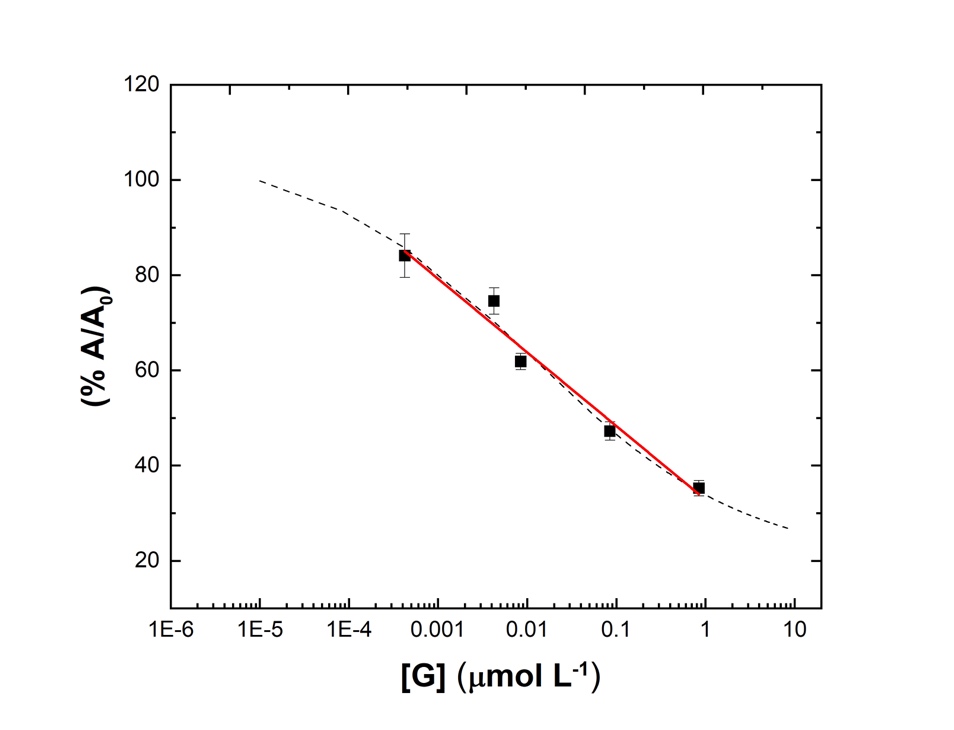


**Fig. S2** The linear range of the sigmoid-shaped curve (0.42 to 850 nmol L^-1^) obtained by analyzing G spiked standard solutions was highlighted in red

**Fig. S3** Representative UV-vis absorption spectra of the BELISA assay, before data elaboration, and an illustrative inset photo of the assay

**LC-MS/MS method**

| **Table S1** MS operative parameters | | | |
| --- | --- | --- | --- |
| **Analyte** | **SRM transition (Da)** | **CE (V)** | **CXP (V)** |
| G | 592.1 🡪 221.1 (q) | 44 | 6.4 |
|  | 592.1 🡪 248.9 (Q) | 38 | 7.1 |
|  | 592.1 🡪 748.1 (q) | 31 | 9.8 |
| (Des-Pyr^1^)-GnRH | 536.4 🡪 110.2 (q) | 32 | 5.7 |
|  | 536.4 🡪 110.2 (Q) | 71 | 5.5 |
|  | 536.4 🡪 934.5 (q) | 29 | 12.2 |


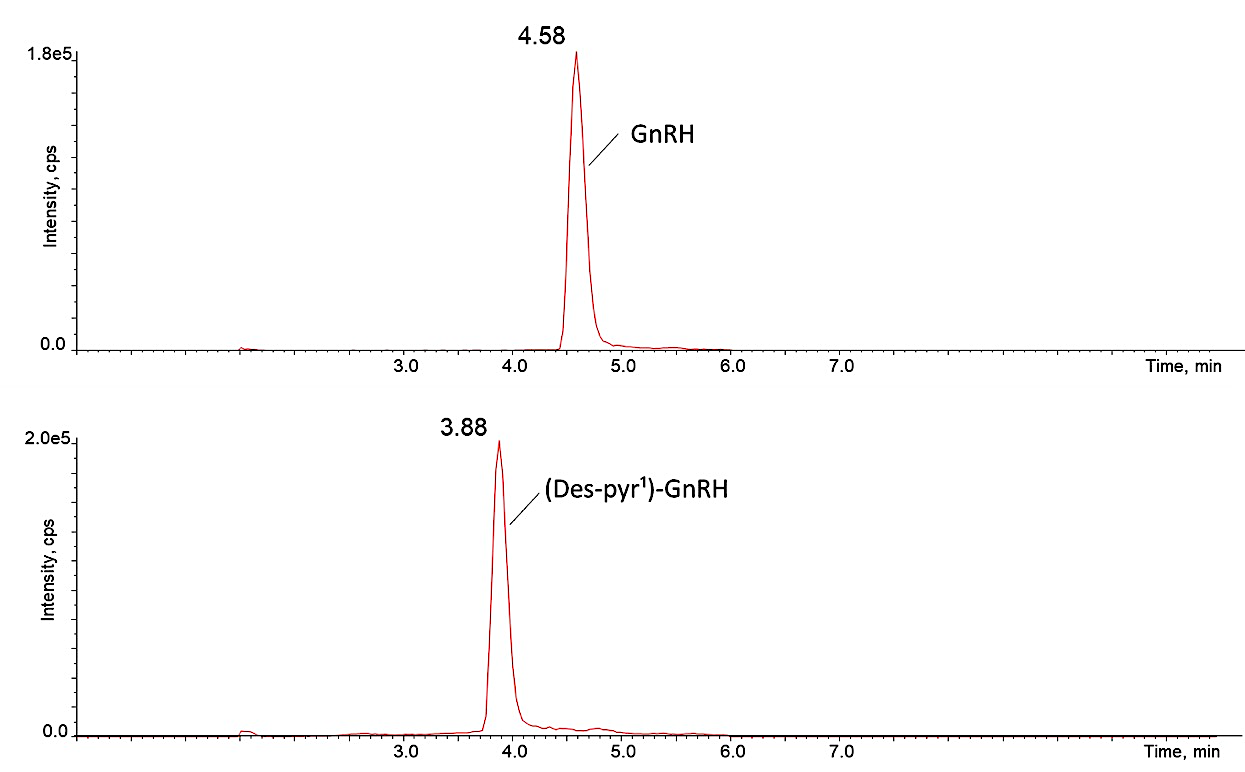


**Fig. S4** Chromatographic profiles of G (aka GnRH) and ISTD (Des-pyr^1^)-GnRH (from top to bottom) and relative retention times
